# Association of the Dietary Approaches to Stop Hypertension, Physical Activity, and Their Combination with Semen Quality: A Cross-Sectional Study

**DOI:** 10.3390/nu12010039

**Published:** 2019-12-22

**Authors:** Anna Danielewicz, Jakub Morze, Mariusz Przybyłowicz, Katarzyna Eufemia Przybyłowicz

**Affiliations:** 1 Department of Human Nutrition, Faculty of Food Sciences, University of Warmia and Mazury in Olsztyn, ul. Słoneczna 45F, 10-718 Olsztyn, Poland; jakub.morze@uwm.edu.pl (J.M.); katarzyna.przybylowicz@uwm.edu.pl (K.E.P.); 2 Department of Cardiology and Cardiac Surgery, School of Medicine, University of Warmia and Mazury in Olsztyn, al. Warszawska 30, 10-082 Olsztyn, Poland; 3 Department of Oncological Gynecology, Provincial Specialist Hospital in Olsztyn, ul. Żołnierska 18, 10-561 Olsztyn, Poland; mprzybylowicz@wp.pl; 4 Center of Gynecology, Endocrinology and Reproductive Medicine Artemida in Olsztyn; ul. Jagiellońska 78, 10-229 Olsztyn, Poland

**Keywords:** DASH diet, physical activity, semen quality, male fertility, sedentary activities, dietary patterns

## Abstract

The influence of individual lifestyle factors is widely described in studies on semen quality. However, their synergistic effect is often neglected. The aim of the study was to examine the association between semen quality and dietary approaches to stop hypertension (DASH) diet, physical activity (PA), and the two separately and in combination. A cross-sectional study was carried out among 207 men aged 20–55. Dietary data were collected by a food frequency questionnaire (FFQ) and analysed according to the DASH scoring index. Physical activity was evaluated by the International Physical Activity Questionnaire. Semen parameters were assessed via the computer-aided semen analysis (CASA). Adherence to the DASH diet was associated with higher sperm count (Δ*_T3-T1_* = 82.1 mln/ej) and concentration (Δ*_T3-T1_* = 24.6 mln/mL). Higher PA was related to higher sperm count (Δ*_T3-T1_* = 69.4 mln/ej), total (Δ*_T3-T1_* = 11.9%), and progressive motility (Δ*_T3-T1_* = 8.5%) and morphology (Δ*_T3-T1_* = 2.8%) in the crude model and remained significant after adjustment. The combination of the DASH diet and PA, was significantly positively associated with sperm count (Δ*_T3-T1_* = 98.1 mln/ej), sperm concentration (Δ*_T3-T1_* = 17.5 mln/mL), total (Δ*_T3-T1_* = 11.8%), and progressive motility (Δ*_T3-T1_* = 10.0%) and morphology (Δ*_T3-T1_* = 3.3%) in both models. Adherence to the DASH diet was related to higher sperm count and concentration, whereas after its combination with physical activity it was also positively correlated with sperm motility and morphology.

## 1. Introduction

In recent years, infertility has been recognized as a rapidly increasing worldwide problem [1]. Childlessness, caused by the male factor, ranges from 20% to 70% and is associated with impaired semen quality [2]. Abnormalities of semen parameters or function, besides anatomical, endocrine, genetic, functional, or immunological disturbances [3], may be caused by nutritional, lifestyle, and environmental factors [4,5,6], and they may occur in men with and without adverse medical history.

Most of the epidemiological studies have assessed the effects of single foods, nutrients and other lifestyle factors on semen parameters [6,7,8,9], whereas their synergistic effect is neglected. Some lifestyle behaviours co-occur together and in combination are more beneficial to health than their cumulative individual effect. Moreover, interventions aimed at multiple-behaviour change may have a potentially greater impact on health than interventions based on a single risk factor [10].

The easiest modifiable lifestyle factors, with the important impact on semen quality, are diet and physical activity. The dietary approaches to stop hypertension (DASH) diet is one of the most well-known dietary strategies, and its effect is often linked with prevention and treatment of hypertension, diabetes, obesity, and coronary artery disease [11]. Moreover, its effects were studied in the context of other diseases [12], including semen quality [13]. The DASH diet can be adapted to any variance in regional diet, which can make lifestyle-related health factors grow more sustainable [14]. The DASH diet reflects a healthy eating pattern holistically, promoting food such as food sources of vitamins, minerals, and antioxidants, and limiting food high in saturated fats, sugar, and sodium. Previous research has indicated that this dietary pattern may be important to maintain proper semen quality [6,13].

Physical activity is one of the health promoting lifestyle factors. However, earlier studies describing a link between physical activity and semen quality provided inconsistent results [15]. Some authors suggested that moderate-to-vigorous physical activity can be related to proper quality sperm parameters, whereas too high or too low [16] physical activity may translate to poorer semen quality. On the other hand, other studies have suggested no association between physical activity (PA) and semen quality [17]. It needs to be underlined that not only the amount but also the type of physical activity is important for male reproductive health [4].

Although we already have some indications about the individual impact of diet [6] and physical activity [4] on semen quality, much uncertainty still exists about the relationship when they co-occur. Thus, the aim of the study was to examine the association between semen quality and the DASH diet, physical activity, both separately and in combination.

## 2. Materials and Methods

### 2.1. Participants

Participants enrolled in this cross-sectional study were men from Northern–Eastern Poland who attended the centre of reproductive medicine in Olsztyn (Poland) or voluntarily joined the study in 2014–2018. The eligibility criteria for the participation in the study was being aged 20–55 years, the absence of a specific clinical condition, willingness to undergo examinations, and the absence of acute or chronic reproductive tract diseases or diagnosed hormonal disorders. Initially, the study population consisted of 541 men who gave their consent to share available results of the semen analysis with the study personnel. However, only 233 (43.1%) men were willing to participate in further stages of the study. During the data verification, subjects were excluded due to missing (*n* = 11) or unreliable (*n* = 15) data. Finally, 207 (38.3%) men were enrolled in the study.

The study protocol was approved by the institutional review board of the Bioethical Committee of the Warmia-Mazury Medical Chamber in Olsztyn (no. 9/2015). All participants gave written informed consent.

### 2.2. Dietary Data Collection

All participants filled out a semi-quantitative 165-food-item food frequency questionnaire (FFQ) validated for the Polish population [18]. During the face-to-face interview, participants were asked about the average portion size and frequency of food consumption ranging as times per day, week, month, or year, and “never”, “I do not know how often”, or “I do not know if I ate”, within a year prior to involvement in the study. Food items from the FFQ were reduced to 23 food groups on the basis of origin and similar nutrient content, and this was used for further analysis.

Adherence to the DASH diet was assessed with the index developed by Günther et al. [19]. DASH diet score is composed of eight food groups. Six components (fruit, including juices; vegetables, including potatoes; meat, poultry, fish, and eggs; nuts, seeds, and legumes; fats and oils; sweets) were on a 10-point scale (0–10) and four components were on a 5-point scale (0–5) (grains: total and high-fibre; dairy products: total and low-fat). Adherence to the DASH diet was based in the overall score, ranging from 0 to 80. Servings intake between a minimum and maximum level were scored proportionally. Individuals received the maximum score if their intakes met the recommendation. In the case of intakes of sweets, fats, and oils, and meat, poultry, fish, and eggs, where lower intakes are favourable, components were reverse-coded and received a maximum score of 10 if their intakes were at or below the target level. The number of each food group servings was assigned on the basis of the energy levels (Table S1) [19,20]. For each individual daily energy intake, we assigned to the closest literature-based energy intake category (1200, 1400, 1600, 1800, 2000, 2600, and 3100 kcal/day) recommended by the National Health Institute [20].

### 2.3. Physical and Sedentary Activity

The procedure for assessing physical activity (PA) and sedentary time were described previously [21]. In brief, PA was evaluated by the validated International Physical Activity Questionnaire (IPAQ)—long version [22]. Participants were asked about average duration (in minutes) and frequency (days) they spent on four PA domains (work-related, transport-related, domestic and gardening (yard), leisure time) during the week prior to the appointment. PA was expressed as a sum of all activities (MET (metabolic equivalent of task) min/week) and as weekly time in which individuals spent on each type of PA intensity (min/week). A sum of the average time per week spent on sitting (at work and home, and while driving) expressed the sedentary time (h/week).

### 2.4. Semen Analysis

Details of the semen sample analysis were described previously [21]. Briefly, samples were collected at the clinic by masturbation. Before analysis, the samples were liquefied for 30 min at 37 °C. Macroscopic examination of raw semen samples was performed manually [23]. Microscopic measurements were determined with the use of computer-aided semen analysis (CASA) by the system composed of a bright-field microscope (Olympus CX41, Tokyo, Japan), a digital camera to capture images (Olympus U-CMAD3, Tokyo, Japan) and professional software (SCA Microptic S.L., Barcelona, Spain). Sperm concentration and motility were determined with a four-chamber GoldCyto slide (10 μm depth) by applying 4 μL of semen, and analysed under phase-contrast optics at ×100 magnification. Morphology of at least 50 spermatozoa was assessed on semen smears stained with Diff-Quick method, using a bright-field optics at 600× magnification. To evaluate abnormal values of semen quality parameters, the WHO [23] cut-off points were used. All analyses were performed by an experienced technician.

### 2.5. Other Measurements

Bodyweight (with accuracy to 0.1 kg) and height (with accuracy to 0.1 m) were measured in light clothing and barefoot using a digital scale and stadiometer, respectively. Body mass index (BMI) was calculated as the ratio of body weight and squared height, and evaluated according to the WHO cut-off points [24]. All participants provided answers regarding their economic status (below average, average, above average), educational level (basic and vocational, intermediate, high), place of residence (village, less than 50,000 citizens, 50,000–100,000 thousand citizens, more than 100,000 citizens), smoking status (current, past, or never), and sleep duration (average hours).

### 2.6. Statistical Analysis

The normality of the data was assessed by the Shapiro–Wilk test. All continuous variables had nonparametric distribution and were presented as the median and interquartile range (IQR). Differences between tertiles of the DASH diet and PA were evaluated using the Kruskal–Wallis test and Dunn’s post-hoc test. Categorical variables were expressed using number and percentage and compared between tertiles using chi-square or Fisher’s exact test.

To extract the participants’ adherence to a low, medium, or high subgroup of the combination of the DASH diet and PA, the matrix based on tertiles distribution of these two factors was created (Scheme S1). Participants with low adherence to the combination of the DASH diet and PA were characterised by a low score of the DASH diet and low or moderate PA, or moderate score of the DASH diet and low PA. Participants with moderate adherence to the combination of the DASH diet and PA were characterised by a high score of the DASH diet and low PA, a high or moderate score of the DASH diet and moderate PA, or moderate or low score of the DASH diet and high PA. Participants with high adherence to the combination of the DASH diet and PA were characterised by a high score of the DASH diet and a high PA.

The association between semen parameters and the DASH diet, PA, and their combination was evaluated by the general linear model. Semen parameters were log-transformed before inclusion in analyses. Linear trends were assessed by assigning the median value (excluding the combination of the DASH diet and PA) within each tertile and modelling this variable continuously. Two adjustment sets were considered: model 1—unadjusted and model 2—adjusted for age, BMI, and energy intake to total energy expenditure ratio (EI/TEE) (as continuous variables), and smoking status (yes/no) and socioeconomic status (low/medium/high) (as categorical variables). Results were presented as backtransformed least square means of semen parameters (and their corresponding standard errors) and their cross-tertile differences (Δ*_T3-T1_*).

Separate models including single components of the DASH diet and PA were fitted to identify those which contributed to the effects observed in main analyses. The applied model was adjusted for age, BMI, EI/TEE ratio (as continuous variables); and smoking status (yes/no) and socioeconomic status (SES) (low/medium/high) (as categorical variables), and respectively for other components of the DASH diet score or a sum of other PA intensities. All *p*-values were two-sided and an alpha level of <0.05 was considered as significant. All statistical tests were performed using STATISTICA software (version 13.1 PL; StatSoft Inc.: Tulsa, USA/Inc: Krakow, Poland)

## 3. Results

Table 1 shows socioeconomic and semen quality characteristics of study participants by tertiles of the DASH diet and PA. The majority of participants, with a median age 26.0 (23.9; 29.0) years and BMI 24.8 (23.1; 26.8), had a normal weight (53.1%), were non-smokers (75.8%), urban residents (73.4%), and had basic or intermediate education (59.9%). In tertiles (T1, T2, T3) of the DASH diet and PA, there were no differences for socioeconomic parameters. None of the semen parameters differed across tertiles of the DASH diet. According to the PA, men in T3 had higher sperm count and concentration, total and progressive motility, as well as morphology compared with men in T1. Significantly more participants with abnormal total and progressive sperm motility were observed in T1 (38.2% and 61.8%, respectively) compared with in T2 (19.1% and 45.6%, respectively) and T3 (18.3% and 42.6%, respectively).

Table S2 shows diet and physical activity characteristics among tertiles of the DASH score and PA. Medians of consumption frequency of vegetables, fruits and nuts, seeds, legumes, and percentage of high-fibre grains and low-fat dairy in the DASH T3 were higher compared with the DASH T1 (3.8 vs. 1.6 times/day, 1.8 vs. 0.6 times/day, 3.7 vs. 0.8 times/week, 32.5% vs. 7.3%, and 80.7% vs. 69.3%, respectively). Median consumption frequencies of meat, fish, eggs, fats and oils, and sweets in the DASH T3 were lower compared with the DASH T1 (3.5 vs. 3.8 times/day, 1.9 vs. 2.7 times/day, and 4.9 vs. 7.2 times/week, respectively). However, none of the consumed food groups were found to differ across PA tertiles. Men in the DASH T3 spent more time on total and moderate PA than men in DASH T2 (205 vs. 116 min/week and 73 vs. 38.5 min/week, respectively), and spent less time sedentary than men in DASH T1 (1.8 vs. 2.9 h/week), but there was no difference in total PA (*p* = 0.598). As expected, we observed the highest total PA and the total time of PA in PA T3, and differences were significant across all tertiles (*p* < 0.001).

Table 2 shows the association between semen parameters and adherence to the DASH diet, PA, and their combination. There were a substantially higher sperm concentration and count across tertiles of DASH diet adherence in the both crude and adjusted model (sperm concentration: Δ*_T3-T1_* = 24.6 mln/ej; sperm count: Δ*_T3-T1_* = 82.1 mln/mL and 85.4 mln/mL in the crude and adjusted model, respectively). However, adherence to the DASH diet was not associated with other semen parameters. A significant positive association was observed in sperm parameters across tertiles of PA. Higher PA was directly associated with sperm count (Δ*_T3-T1_* = 69.4 mln/ej, *p*_trend_ = 0.043), total motility (Δ*_T3-T1_* = 11.9%, *p*_trend_ < 0.001), progressive motility (Δ*_T3-T1_* = 8.5%, *p*_trend_ < 0.001), and morphology (Δ*_T3-T1_* = 2.8%, *p*_trend_ = 0.003) in the crude model. Except for sperm count, these effects remained significant after adjusting for age, BMI, EI/TEE, smoking status, and SES. A key point of the analysis was to compare sperm parameters across tertiles of the DASH diet and PA combination. Both in crude and adjusted models, positive cross-tertile trends were observed in sperm concentration (Δ*_T3-T1_* = 17.5 mln/ej; *p*_trend_ = 0.016), sperm count (Δ*_T3-T1_* = 98.1 mln/mL; *p*_trend_ = 0.036), total (Δ*_T3-T1_* = 11.8%; *p*_trend_ = 0.002), and progressive motility (Δ*_T3-T1_* = 10.0%; *p*_trend_ = 0.003) and morphology (Δ*_T3-T1_* = 3.3%; *p*_trend_ = 0.004).

The results of the analysis for individual DASH diet components (after adjustment for multiple confounders) (Table 3) indicated a negative association between grains and sperm concentration (β = −0.162; *p* = 0.028) and total motility (β = −0.162; *p* = 0.030), as well as dairy and sperm concentration (β = −0.147; *p* = 0.037). A significant positive association was found between low-fat dairy and sperm volume (β = 0.186; *p* = 0.009); nuts, seeds, and legumes and sperm concentration (β = 0.158; *p* = 0.033); and fats and oils and sperm count (β = 0.188; *p* = 0.011). Sweets scoring was also positively associated with sperm concentration (β = 0.190; *p* = 0.010), but reverse coding needs to be taken into account. We also evaluated the link between semen parameters and each level of PA intensity (Table 3). The results indicate that vigorous PA was positively associated with sperm count (β = 0.188; *p* = 0.009), total (β = 0.182; *p* = 0.009) and progressive (β = 0.182; *p* = 0.010) motility, and morphology (β = 0.195; *p* = 0.007). Moderate PA was positively associated with total sperm motility (β = 0.143; *p* = 0.049).

## 4. Discussion

In this study, we investigated the association between semen quality and DASH diet, PA, and their combination. We found that greater adherence to the DASH diet was associated with higher sperm count and concentration, whereas higher PA level was associated with better total and progressive motility and morphology. To our best knowledge, this is the first study assessing the cluster of diet and PA in relation to semen quality. Our study provides evidence that assessing the effect of a healthy diet combined with a higher level of PA may be a better indicator of semen quality than evaluation of individual factors in separation.

Studies assessing the effect of healthy dietary patterns, regardless of whether they were data- or hypothesis-driven, presented a positive relationship with sperm quality [25]. Healthy dietary patterns, including the DASH diet, correlated positively with the intake of foods or nutrients, which have a favourable effect on maintaining or improving semen quality [26]. In our study, we did not observe differences in semen parameters across tertiles of the DASH diet. However, higher adherence to this diet was associated with higher sperm count and concentration. Additionally, analysis of single components of the DASH diet showed that sperm concentration was positively correlated with nuts, seeds, and legumes and inversely with sweets, total dairy, and total grains. Sperm volume and count were positively associated with low-fat dairy and fats and oils, respectively, whereas total sperm motility was inversely associated with total grains. Our results are partially consistent with Cutillas-Tolin et al. [27] where higher adherence to the DASH diet was associated with higher sperm concentration, total sperm count, and total motile sperm count in healthy young Spanish students. Some similarities may also be found with observations made by Efrat et al. [13], where higher adherence to the DASH diet was associated with higher sperm concentration and morphology among men attending an Israeli fertility centre.

The DASH diet, similar to the Mediterranean diet and prudent dietary style, assumes a higher intake of fruit, vegetables, whole grains, and legumes. These food groups are positively associated with sperm count, concentration [28], sperm motility [29,30], and morphology [28]. As a naturally rich source of vitamins, minerals, and polyphenols, the DASH diet may be perceived as antioxidative and anti-inflammatory. The antioxidants in male reproductive health may be positively associated with semen parameters [31,32,33], fertilization rate [34], decreasing oxidative damage to spermatozoa [35,36], DNA maintenance, transfer RNA, protein synthesis [37], and the reduction of the negative effects of inflammation [36,38]. Moreover, the DASH diet recommends a reduction in sweets, meat, eggs, and fat consumption that, due to the content of sugars, saturated/trans fatty acids, and sodium, may adversely affect sperm count [28], concentration [39,40], sperm motility [29,40,41], and morphology [40,41].

In our study, semen parameters differed across PA tertiles and were positively correlated with PA. Analysis of the impact of individual PA components on semen parameters indicated that the obtained results were mostly affected by vigorous PA. In our study, the type of PA that corresponded to this effect was work-related activity. This result suggests that a high cumulative effect of PA from the whole day could be as important as recreational PA.

Ibanez-Perez et al. [4] in their meta-analysis revealed that moderate or high recreational PA could have a beneficial influence on semen quality. Authors also highlighted that different types of PA did not modify that association. On the other hand, elite PA was correlated with a decline in volume, concentration, total motility, and morphology [4] and affects sperm DNA fragmentation [42]. However, some studies present that PA was not related to semen quality in young men and those from infertile couples [4,17,43,44]. Nevertheless, the promotion of moderate-to-vigorous PA may improve semen parameters, sperm DNA integrity, impaired seminal markers of inflammation, and oxidative stress [45,46]. Interestingly, the impact of different intensities showed an inverted U-shape association between PA and semen quality [47,48]. Also, other studies supported this finding, suggesting poorer semen quality in sedentary [7,16,49] or highly active men [42] compared with recreationally or moderate physically active men.

The possible mechanisms through which PA might exert its effect on semen are oxidative stress, impaired hypothalamus-pituitary-testicular (HPT) axis function, and hormonal imbalance [50,51,52]. Exercise naturally increases oxygen consumption, resulting in a general increase in reactive oxygen species (ROS) production. With increased exercise intensity, the body can no longer eliminate the increasing ROS concentration, which leads to oxidative stress and damage of cellular components [50,51]. These effects can be explained by the so-called hormesis theory. Regular moderate PA improves antioxidant enzyme activity and resistance to oxidative stress. Simultaneously, forceful exercise results in an increase of oxidative stress, ROS, and oxidative damage, whereas the activity of antioxidant enzymes and the capacity of oxidative damage repair are impaired [50]. The impact of PA on HPT axis functioning can be miscellaneous and depends on activity levels [52,53]. A moderate PA could be related to higher follicle-stimulating hormone (FSH), luteinizing hormone (LH), and testosterone (T) level in comparison to the sedentary controls [54]. High-intensity exercise may lead to a decrease in plasma LH, FSH, and T concentration, as well as sperm parameters [55]. The suppression of the HPT axis could be caused by decreased production of gonadotropin-releasing hormone and a reduced response of gonadotropins during acute prolonged physical exercise [53,56].

The major strength of this study was the use of validated questionnaires for assessing food consumption and PA. Also, the FFQ measures food intake during the past 12 months, which minimises risk of misclassification of the DASH score and overlaps with spermatogenesis time for the taken sample. Additionally, the use of hypothesis-based dietary patterns more closely reflects real food consumption, and it could be easily applicable by other researchers or dietitians and physicians in their practice.

However, some limitations of the study should be acknowledged. First, the nature of the cross-sectional study limited the identification of causal relationships between diet, PA, and semen quality. Secondly, we assessed the semen quality based at a one-time point, which does not consider the within-person variability in semen parameters. Third, the usage of the questionnaire to assess PA could be a source of bias, whereas the use of accelerometers is a recommended method to objectively measure PA. Our study measured the semen condition only in the context of six parameters and did not include the viability, acrosomal reactivity, and spermatozoa kinematics parameters, which are useful for evaluating semen quality and its fertilization capacity. Also, the number of participants with low PA was relatively small, which did not allow for the selection of a subgroup strongly adherent to a sedentary lifestyle.

## 5. Conclusions

Adherence to the DASH diet was related to higher sperm count and concentration, whereas their combination with physical activity was also positively correlated with sperm motility and morphology. Furthermore, we observed that daily physical activity was firmly associated with semen parameters. Following our novel findings, we would recommend the use of the DASH diet and physical activity together as a better indicator of semen quality compared with both factors analysed separately. However, our findings need to be confirmed by further prospective observational and clinical studies. Because the management of infertility involves many different treatment strategies, cooperation between physicians, dieticians, and public health educators might be of great importance.

## Figures and Tables

**Table 1:** Socioeconomics and semen quality characteristics among tertiles of dietary approaches to stop hypertension (DASH) score and physical activity.

Variable	Total	DASH			*p*	Physical Activity			*p*
		T1	T2	T3		T1	T2	T3	
*n*	207	62	75	70		68	68	71	
DASH score	34–76	34–45	46–54	55–76		47 (40; 51)	49.5 (41; 56)	47 (42; 53)	0.377
Age (years)	26.0 (23.9; 29.0)	26.0 (24.2; 30.0)	26.0 (23.3; 29.0)	25.2 (24.0; 28.3)	0.651	26.0 (24.2; 32.0)	26.0 (23.0 27.5)	26.0 (23.6; 28.3)	0.058
BMI (kg/m^2^)	24.8 (23.1; 26.8)	24.8 (22.7; 27.0)	24.8 (23.2; 26.9)	24.9 (23.7; 26.5)	0.986	24.9 (23.4; 27.1)	24.6 (22.6; 26.1)	25.2 (23.7; 26.9)	0.209
Overweight or obese, *n* (%)	97 (46.9)	30 (48.4)	34 (45.3)	33 (47.1)	0.937	33 (48.5)	28 (41.2)	36 (50.7)	0.502
Urban residence, *n* (%)	152 (73.4)	42 (67.7)	56 (77.1)	54 (77.1)	0.454	55 (80.9)	49 (72.1)	48 (67.6)	0.198
Higher education, *n* (%)	83 (40.1)	19 (30.7)	30 (40.0)	34 (48.6)	0.111	29 (42.7)	28 (41.2)	26 (36.6)	0.750
Current smokers, *n* (%)	50 (24.2)	19 (30.6)	19 (25.3)	12 (17.1)	0.186	16 (23.5)	14 (20.6)	20 (28.2)	0.574
Energy intake (kcal/day)	2058.7 (1591.6; 2418.8)	2079.3 (1485.2; 2381.7)	2081.7 (1602.9; 2410.4)	2002.6 (1625.1; 2514.8)	0.817	1794.8 (1521.6; 2211.6)	2050.1 (1576.0; 2842.4)	2100.0 (1685.9; 2514.8)	0.118
Semen parameters									
Volume (mL)	4.0 (2.7; 5.0)	4.2 (3.1; 5.2)	3.8 (2.5; 5.0)	3.8 (2.5; 5.0)	0.257	3.1 (2.8; 4.9)	4.0 (3.0; 5.0)	4.0 (2.5; 5.4)	0.708
Abnormal volume ^1^	8 (3.9)	4 (6.5)	4 (5.3)	0 (0.0)	0.113	2 (2.9)	2 (2.9)	4 (5.6)	0.634
Sperm concentration (mln/mL)	27.9 (10.3; 63.6)	20.8 (9.8; 41.7)	30.6 (12; 61.4)	34.7 (10.6; 78.1)	0.087	20.4 (7.7; 44.7) ^a^	31.1 (11.2; 64.2)	33.2 (14.4; 78.5) ^a^	0.029
Abnormal sperm concentration ^1^	68 (32.9)	26 (41.9)	21 (28.0)	21 (30.0)	0.185	27 (39.7)	22 (32.4)	19 (26.8)	0.266
Sperm count (mln/ejaculate)	102.9 (37.9; 219)	74.6 (31.3; 165.7)	115.7 (37.9; 201)	121.8 (45.8; 295.2)	0.153	74.1 (26.9; 162.9) ^a^	108.9 (49.4; 231.2)	124.5 (48.4; 306.3) ^a^	0.019
Abnormal sperm count ^1^	53 (25.6)	18 (29.0)	20 (26.7)	15 (21.4)	0.586	24 (35.3)	16 (23.5)	13 (18.3)	0.064
Total motility (%)	52.7 (39.8; 65.1)	50.5 (39.7; 62.5)	52.1 (40.8; 65.3)	54.9 (39.8; 67.0)	0.481	47.9 (32.8; 58.6) ^a^	54.6 (42.3; 62.9)	57.4 (47.5; 73.5) ^a^	<0.001
Abnormal total motility ^1^	52 (25.1)	16 (25.8)	18 (24.0)	18 (25.7)	0.961	26 (38.2)	13 (19.1)	13 (18.3)	0.010
Progressive motility (%)	32.1 (21.7; 43.6)	31.0 (20.9; 40.5)	31.3 (21.7; 41.2)	34.1 (22.1; 45.2)	0.541	26.8 (16; 36.1) ^a, b^	33.0 (22.5; 44.1) ^a^	36.1 (24.4; 49.0) ^b^	0.002
Abnormal progressive motility ^1^	103 (49.8)	35 (56.5)	40 (53.3)	28 (40.0)	0.125	42 (61.8)	31 (45.6)	30 (42.6)	0.049
Morphology (% of normal)	7.0 (4.0; 12.0)	6.0 (4.0; 9.0)	8.0(4.0; 13.0)	8.0 (4.0; 13.0)	0.554	6.0 (2.1; 9.5) ^a^	8.0 (4.0; 12.5)	8.2 (4.0; 14.0) ^a^	0.010
Abnormal morphology ^1^	43 (20.8)	13 (21.0)	16 (21.3)	14 (20.0)	0.980	20 (29.4)	11 (16.2)	12 (16.9)	0.100

Data are presented as median and interquartile range (IQR) for continuous variables and number and (%) categorical variables. DASH: diet approaches to stop hypertension, BMI: body mass index. ^1^ Compared to group with normal semen parameters. *p*-values for continuous variables were derived from the Kruskal–Wallis test (^a, b^ Dunn post-hoc test presented differences between pairs of tertiles) and categorical variables were derived from the χ^2^ test.

**Table 2:** DASH, physical activity, and their combination in relation to semen parameters.

Variable	DASH				Δ*_T3-T1_*	*p*_trend_	Physical Activity			Δ*_T3-T1_*	*p*_trend_	DASH and Physical Activity			Δ*_T3-T1_*	*p*_trend_
	T1		T2	T3			T1	T2	T3			T1	T2	T3		
*n*	62		75	70			68	68	71			68	113	26		
Sperm volume (mL)																
Crude	4.3 (0.2)		3.7 (0.2)	4.1 (0.2)	0.2	0.617	3.8 (0.2)	4.0 (0.2)	4.2 (0.3)	0.4	0.252	4.0 (0.2)	3.9 (0.2)	4.6 (0.5)	0.6	0.271
Model 1	4.3 (0.2)		3.7 (0.2)	4.0 (0.2)	0.3	0.598	3.8 (0.2)	3.9 (0.2)	4.1 (0.2)	0.3	0.277	3.9 (0.2)	3.8 (0.2)	4.6 (0.4)	0.7	0.288
Sperm count (mln/ej)																
Crude	136.1 (20.3)		162.6 (20.9)	218.2 (29.3)	82.1	0.018	138.7 (23.8)	172.1 (21.8)	208.1 (26.4)	69.4	0.043	124.9 (18.5)	191.4 (20.1)	222.5 (47.0)	97.6	0.014
Model 1	135.9 (26.7)		162.6 (25.2)	221.3 (26.9)	85.4	0.016	137.0 (26.7)	169.3 (27.2)	203.2 (25.7)	66.2	0.057	125.4 (25.9)	189.1 (21.2)	222.5 (41.4)	98.1	0.016
Sperm concentration (mln/mL)																
Crude	36.2 (5.5)		46.1 (5.3)	60.8 (7.9)	24.6	0.008	38.3 (5.9)	51.6 (7.1)	54.1 (6.3)	15.8	0.101	33.9 (4.6)	55.7 (5.8)	51.9 (9.3)	18.0	0.032
Model 1	37.2 (7.1)		46.2 (6.7)	61.8 (7.2)	24.6	0.009	38.3 (7.1)	50.5 (7.3)	54.2 (6.9)	15.9	0.107	34.0 (6.9)	55.8 (5.7)	51.5 (11.0)	17.5	0.036
Total motility (%)																
Crude	51.0 (2.2)		52.1 (2.0)	54.0 (2.2)	3.0	0.314	45.9 (2.0)	53.3 (1.9)	57.8 (2.2)	11.9	<0.001	47.9 (1.9)	53.5 (1.6)	59.8 (4.1)	11.9	0.002
Model 1	51.8 (2.3)		53.1 (2.2)	55.1 (2.4)	3.3	0.301	46.7 (2.2)	53.7 (2.3)	58.9 (2.2)	12.2	<0.001	48.7 (2.2)	54.6 (1.8)	60.5 (3.6)	11.8	0.002
Progressive motility (%)																
Crude	32.0 (1.9)		31.6 (1.6)	34.1. (1.9)	2.1	0.411	27.7 (1.7)	33.2 (1.6)	36.2 (1.8)	8.5	<0.001	29.2 (1.7)	33.1 (1.4)	39.4 (3.3)	10.2	0.003
Model 1	32.8 (2.0)		32.3 (1.9)	35.0 (2.0)	2.2	0.394	28.4 (1.9)	33.5 (1.9)	37.5 (1.8)	9.1	<0.001	29.8 (1.9)	34.1 (1.5)	39.8 (3.0)	10.0	0.003
Morphology (%)																
Crude	7.5 (0.6)		8.5 (0.7)	8.6 (0.7)	1.1	0.267	6.6 (0.6)	8.6 (0.7)	9.4 (0.7)	2.8	0.003	6.8 (0.6)	8.7 (0.5)	10.0 (1.4)	3.2	0.004
Model 1	7.5 (0.6)		8.5 (0.7)	8.6 (0.7)	1.1	0.309	6.7 (0.7)	8.5 (0.7)	9.7 (0.7)	3.0	0.002	6.9 (0.7)	8.9 (0.6)	10.2 (1.1)	3.3	0.004

Data are presented as least square means and standard error (SE). Reference groups are participants from a group of normal semen parameters (according to WHO 2010 recommendation). Crude: unadjusted; model 1: adjusted for age, BMI, and energy intake to total energy expenditure ratio (EI/TEE) ratio (as continuous variables), and smoking status (yes/no) and socioeconomic status (low/medium/high) (as categorical variables).

**Table 3:** Association of semen parameters by individual DASH diet components and physical activity components (*n* = 207).

Variable	Semen Volume		Sperm Concentration		Sperm Count		Total Motility		Progressive Motility		Morphology	
	β (SE) *	*p*	β (SE) *	*p*	β (SE) *	*p*	β (SE) *	*p*	β (SE) *	*p*	β (SE) *	*p*
DASH components ^a^												
Grains, total (servings/day)	0.105 (0.075)	0.165	−0.162 (0.073)	0.028	−0.072 (0.075)	0.338	−0.162 (0.074)	0.030	−0.137 (0.074)	0.066	−0.082 (0.075)	0.275
High fibre grains (% of daily servings)	0.115 (0.077)	0.135	−0.011 (0.076)	0.880	0.068 (0.076)	0.375	0.111 (0.077)	0.147	0.087 (0.077)	0.258	0.076 (0.769)	0.322
Vegetables (servings/day)	−0.038 (0.072)	0.596	0.021 (0.071)	0.763	−0.023 (0.071)	0.744	−0.072 (0.071)	0.309	−0.039 (0.071)	0.588	−0.068 (0.071)	0.337
Fruit (servings/day)	−0.076 (0.073)	0.300	0.022 (0.072)	0.762	0.006 (0.073)	0.933	0.117 (0.073)	0.109	0.086 (0.073)	0.240	0.035 (0.073)	0.631
Dairy, total (servings/day)	0.104 (0.071)	0.146	−0.147 (0.070)	0.037	−0.100 (0.071)	0.158	−0.015 (0.071)	0.830	0.031 (0.071)	0.669	−0.007 (0.072)	0.917
Low-fat dairy (% of daily servings)	0.186 (0.071)	0.009	0.044 (0.072)	0.543	0.139 (0.071)	0.053	−0.078 (0.072)	0.280	0.009 (0.072)	0.904	−0.083 (0.072)	0.251
Meat, poultry, fish, eggs (servings/day)	−0.076 (0.070)	0.281	0.035 (0.070)	0.618	0.005 (0.070)	0.947	0.074 (0.070)	0.291	0.046 (0.070)	0.515	0.032 (0.070)	0.653
Nuts, seeds, legumes (servings/week)	−0.030 (0.075)	0.693	0.158 (0.074)	0.033	0.101 (0.074)	0.176	0.117 (0.074)	0.119	0.122 (0.074)	0.102	0.086 (0.075)	0.251
Fats, oils (servings/day)	0.052 (0.074)	0.488	0.133 (0.073)	0.073	0.188 (0.073)	0.011	−0.015 (0.074)	0.840	−0.039 (0.074)	0.600	0.053 (0.074)	0.479
Sweets (servings/week)	−0.096 (0.074)	0.198	0.190 (0.073)	0.010	0.097 (0.074)	0.189	0.074 (0.074)	0.320	0.005 (0.074)	0.946	0.091 (0.074)	0.223
Physical activity ^b^												
Vigorous (MET-min/week)	0.060 (0.072)	0.406	0.132 (0.072)	0.070	0.188 (0.071)	0.009	0.182 (0.069)	0.009	0.182 (0.070)	0.010	0.195 (0.071)	0.007
Moderate (MET-min/week)	0.144 (0.075)	0.055	0.099 (0.075)	0.192	0.107 (0.074)	0.152	0.143 (0.072)	0.049	0.106 (0.073)	0.146	0.039 (0.074)	0.602
Walking (MET-min/week)	0.068 (0.074)	0.364	−0.080 (0.075)	0.282	−0.026 (0.074)	0.723	0.109 (0.072)	0.133	0.100 (0.072)	0.168	−0.010 (0.074)	0.893

Data are presented as β coefficients and standard errors (SE). * Adjusted for age, BMI, and EI/TEE ratio (as continuous variables), and smoking status (yes/no) and socioeconomic status (low/medium/high) (as categorical variables). ^a^ Other components of DASH diet score or ^b^ sum of other physical activity intensity. MET: metabolic equivalent of task.

## Supplementary Materials

The following are available online at https://www.mdpi.com/2072-6643/12/1/39/s1: Table S1: List of components of the DASH score and their description; Table S2: Diet and physical activity characteristics among tertiles of DASH score and physical activity; Scheme S1: Matrix of the DASH diet and physical activity tertile distribution.

Supplementary File 1

Supplementary File 2

## References

[1] Agarwal A., Mulgund A., Hamada A., Chyatte M.R. (2015). A unique view on male infertility around the globe. Reprod. Biol. Endocrinol..

[2] Inhorn M.C., Patrizio P. (2015). Infertility around the globe: New thinking on gender, reproductive technologies and global movements in the 21st century. Hum. Reprod. Updat..

[3] Zegers-Hochschild F., Adamson G.D., Dyer S., Racowsky C., de Mouzon J., Sokol R., Rienzi L., Sunde A., Schmidt L., Cooke I.D. (2017). The International Glossary on Infertility and Fertility Care, 2017. Fertil. Steril..

[4] Ibanez-Perez J., Santos-Zorrozua B., Lopez-Lopez E., Matorras R., Garcia-Orad A. (2019). An update on the implication of physical activity on semen quality: A systematic review and meta-analysis. Arch. Gynecol. Obstet..

[5] Ilacqua A., Izzo G., Emerenziani G.P., Baldari C., Aversa A. (2018). Lifestyle and fertility: The influence of stress and quality of life on male fertility. Reprod. Biol. Endocrinol..

[6] Salas-Huetos A., James E.R., Aston K.I., Jenkins T.G., Carrell D.T. (2019). Diet and sperm quality: Nutrients, foods and dietary patterns. Reprod. Biol..

[7] Gaskins A.J., Mendiola J., Afeiche M., Jorgensen N., Swan S.H., Chavarro J.E. (2015). Physical activity and television watching in relation to semen quality in young men. Br. J. Sports Med..

[8] Ricci E., Noli S., Ferrari S., La Vecchia I., Cipriani S., De Cosmi V., Somigliana E., Parazzini F. (2018). Alcohol intake and semen variables: Cross-sectional analysis of a prospective cohort study of men referring to an Italian Fertility Clinic. Andrology.

[9] Jensen T.K., Andersson A.M., Skakkebaek N.E., Joensen U.N., Blomberg Jensen M., Lassen T.H., Nordkap L., Olesen I.A., Hansen A.M., Rod N.H. (2013). Association of sleep disturbances with reduced semen quality: A cross-sectional study among 953 healthy young Danish men. Am. J. Epidemiol..

[10] Noble N., Paul C., Turon H., Oldmeadow C. (2015). Which modifiable health risk behaviours are related? A systematic review of the clustering of Smoking, Nutrition, Alcohol and Physical activity (‘SNAP’) health risk factors. Prev. Med..

[11] Suri S., Kumar V., Kumar S., Goyal A., Tanwar B., Kaur J., Kaur J. (2019). DASH dietary pattern: A treatment for non-communicable diseases. Curr. Hypertens. Rev..

[12] Challa H.J., Tadi P., Uppaluri K.R. (2019). DASH Diet (Dietary Approaches to Stop Hypertension).

[13] Efrat M., Stein A., Pinkas H., Unger R., Birk R. (2018). Dietary patterns are positively associated with semen quality. Fertil. Steril..

[14] Nathenson P. (2017). The DASH diet: A cultural adaptation. Nursing.

[15] Hayden R.P., Flannigan R., Schlegel P.N. (2018). The Role of Lifestyle in Male Infertility: Diet, Physical Activity, and Body Habitus. Curr. Urol. Rep..

[16] Priskorn L., Jensen T.K., Bang A.K., Nordkap L., Joensen U.N., Lassen T.H., Olesen I.A., Swan S.H., Skakkebaek N.E., Jorgensen N. (2016). Is Sedentary Lifestyle Associated With Testicular Function? A Cross-Sectional Study of 1,210 Men. Am. J. Epidemiol..

[17] Minguez-Alarcon L., Chavarro J.E., Mendiola J., Gaskins A.J., Torres-Cantero A.M. (2014). Physical activity is not related to semen quality in young healthy men. Fertil. Steril..

[18] Wadolowska L. (2005). Walidacja kwestionariusza częstotliwości spożycia żywności—FFQ. Ocena powtarzalności [Validation of food frequency questionnaire—FFQ. Reproducibility assessment]. Bromat. Chem. Toksykol..

[19] Günther A.L.B., Liese A.D., Bell R.A., Dabelea D., Lawrence J.M., Rodriguez B.L., Standiford D.A., Mayer-Davis E.J. (2009). Association Between the Dietary Approaches to Hypertension Diet and Hypertension in Youth With Diabetes Mellitus. Hypertension.

[20] National Heart, Lung and Blood Institute of National Institute of Health DASH Eating Plan. https://www.nhlbi.nih.gov/health-topics/dash-eating-plan.

[21] Danielewicz A., Przybylowicz K.E., Przybylowicz M. (2018). Dietary Patterns and Poor Semen Quality Risk in Men: A Cross-Sectional Study. Nutrients.

[22] Craig C.L., Marshall A.L., Sjostrom M., Bauman A.E., Booth M.L., Ainsworth B.E., Pratt M., Ekelund U., Yngve A., Sallis J.F. (2003). International physical activity questionnaire: 12-country reliability and validity. Med. Sci Sports Exerc..

[23] World Health Organization (2010). WHO Laboratory Manual for the Examination and Processing of Human Semen.

[24] World Health Organization Global database on Body Mass Index: BMI Classification. http://apps.who.int/bmi/index.jsp?introPage=intro_3.html.

[25] Arab A., Rafie N., Mansourian M., Miraghajani M., Hajianfar H. (2018). Dietary patterns and semen quality: A systematic review and meta-analysis of observational studies. Andrology.

[26] Salas-Huetos A., Bullo M., Salas-Salvado J. (2017). Dietary patterns, foods and nutrients in male fertility parameters and fecundability: A systematic review of observational studies. Hum. Reprod. Updat..

[27] Cutillas-Tolin A., Adoamnei E., Navarrete-Munoz E.M., Vioque J., Monino-Garcia M., Jorgensen N., Chavarro J.E., Mendiola J., Torres-Cantero A.M. (2019). Adherence to diet quality indices in relation to semen quality and reproductive hormones in young men. Hum. Reprod..

[28] Mendiola J., Torres-Cantero A.M., Moreno-Grau J.M., Ten J., Roca M., Moreno-Grau S., Bernabeu R. (2009). Food intake and its relationship with semen quality: A case-control study. Fertil. Steril..

[29] Eslamian G., Amirjannati N., Rashidkhani B., Sadeghi M.R., Hekmatdoost A. (2012). Intake of food groups and idiopathic asthenozoospermia: A case-control study. Hum. Reprod..

[30] Gaskins A.J., Colaci D.S., Mendiola J., Swan S.H., Chavarro J.E. (2012). Dietary patterns and semen quality in young men. Hum. Reprod..

[31] Minguez-Alarcon L., Mendiola J., Lopez-Espin J.J., Sarabia-Cos L., Vivero-Salmeron G., Vioque J., Navarrete-Munoz E.M., Torres-Cantero A.M. (2012). Dietary intake of antioxidant nutrients is associated with semen quality in young university students. Hum. Reprod..

[32] Ross C., Morriss A., Khairy M., Khalaf Y., Braude P., Coomarasamy A., El-Toukhy T. (2010). A systematic review of the effect of oral antioxidants on male infertility. Reprod. Biomed. Online.

[33] Zareba P., Colaci D.S., Afeiche M., Gaskins A.J., Jorgensen N., Mendiola J., Swan S.H., Chavarro J.E. (2013). Semen quality in relation to antioxidant intake in a healthy male population. Fertil. Steril..

[34] Li M.C., Chiu Y.H., Gaskins A.J., Minguez-Alarcon L., Nassan F.L., Williams P.L., Petrozza J., Hauser R., Chavarro J.E. (2019). Men’s Intake of Vitamin C and beta-Carotene Is Positively Related to Fertilization Rate but Not to Live Birth Rate in Couples Undergoing Infertility Treatment. J. Nutr..

[35] Smits R.M., Mackenzie-Proctor R., Yazdani A., Stankiewicz M.T., Jordan V., Showell M.G. (2019). Antioxidants for male subfertility. Cochrane Database Syst. Rev..

[36] Moretti E., Cosci I., Spreafico A., Serchi T., Cuppone A.M., Collodel G. (2009). Semen characteristics and inflammatory mediators in infertile men with different clinical diagnoses. Int. J. Androl..

[37] Molloy A.M. (2012). Genetic aspects of folate metabolism. Sub-cel. Biochem..

[38] La Vignera S., Condorelli R.A., Vicari E., Tumino D., Morgia G., Favilla V., Cimino S., Calogero A.E. (2013). Markers of semen inflammation: Supplementary semen analysis?. J. Reprod. Immunol..

[39] Attaman J.A., Toth T.L., Furtado J., Campos H., Hauser R., Chavarro J.E. (2012). Dietary fat and semen quality among men attending a fertility clinic. Hum. Reprod..

[40] Liu C.Y., Chou Y.C., Chao J.C., Hsu C.Y., Cha T.L., Tsao C.W. (2015). The Association between Dietary Patterns and Semen Quality in a General Asian Population of 7282 Males. PLoS ONE.

[41] Chiu Y.H., Afeiche M.C., Gaskins A.J., Williams P.L., Mendiola J., Jorgensen N., Swan S.H., Chavarro J.E. (2014). Sugar-sweetened beverage intake in relation to semen quality and reproductive hormone levels in young men. Hum. Reprod..

[42] Vaamonde D., Algar-Santacruz C., Abbasi A., Garcia-Manso J.M. (2018). Sperm DNA fragmentation as a result of ultra-endurance exercise training in male athletes. Andrologia.

[43] Ibañez-Perez J., Santos-Zorrozua B., Lopez-Lopez E., Irazusta J., Prieto B., Aparicio V., Corcostegui B., Gracia-Orad Á., Matorras R. (2019). Impact of physical activity on semen quality among men from infertile couples. Eur. J. Obstet. Gynecol. Reprod. Biol..

[44] Wise L.A., Cramer D.W., Hornstein M.D., Ashby R.K., Missmer S.A. (2011). Physical activity and semen quality among men attending an infertility clinic. Fertil. Steril..

[45] Hajizadeh Maleki B., Tartibian B., Chehrazi M. (2019). Effects of Aerobic, Resistance, and Combined Exercise on Markers of Male Reproduction in Healthy Human Subjects: A Randomized Controlled Trial. J. Strength and Cond. Res..

[46] Hajizadeh Maleki B., Tartibian B. (2017). Moderate aerobic exercise training for improving reproductive function in infertile patients: A randomized controlled trial. Cytokine.

[47] Parn T., Grau Ruiz R., Kunovac Kallak T., Ruiz J.R., Davey E., Hreinsson J., Wanggren K., Salumets A., Sjostrom M., Stavreus-Evers A. (2015). Physical activity, fatness, educational level and snuff consumption as determinants of semen quality: Findings of the ActiART study. Reprod. Biomed. Online.

[48] Hajizadeh Maleki B., Tartibian B., Eghbali M., Asri-Rezaei S. (2013). Comparison of seminal oxidants and antioxidants in subjects with different levels of physical fitness. Andrology.

[49] Lalinde-Acevedo P.C., Mayorga-Torres B.J.M., Agarwal A., du Plessis S.S., Ahmad G., Cadavid A.P., Cardona Maya W.D. (2017). Physically Active Men Show Better Semen Parameters than Their Sedentary Counterparts. Int. J. Fertil. Steril..

[50] Pingitore A., Lima G.P., Mastorci F., Quinones A., Iervasi G., Vassalle C. (2015). Exercise and oxidative stress: Potential effects of antioxidant dietary strategies in sports. Nutrition.

[51] Gomes M., Freitas M.J., Fardilha M. (2015). Physical Activity, Exercise, and Mammalian Testis Function: Emerging Preclinical Protein Biomarker and Integrative Biology Insights. Omics J. Integr. Biol..

[52] Di Luigi L., Romanelli F., Sgro P., Lenzi A. (2012). Andrological aspects of physical exercise and sport medicine. Endocrine.

[53] Sansone A., Sansone M., Vaamonde D., Sgro P., Salzano C., Romanelli F., Lenzi A., Di Luigi L. (2018). Sport, doping and male fertility. Reprod. Biol. Endocrinol..

[54] Vaamonde D., Da Silva-Grigoletto M.E., Garcia-Manso J.M., Barrera N., Vaamonde-Lemos R. (2012). Physically active men show better semen parameters and hormone values than sedentary men. Eur. J. Appl. Physiol..

[55] Safarinejad M.R., Azma K., Kolahi A.A. (2009). The effects of intensive, long-term treadmill running on reproductive hormones, hypothalamus-pituitary-testis axis, and semen quality: A randomized controlled study. J. Endocrinol..

[56] Cano Sokoloff N., Misra M., Ackerman K.E. (2016). Exercise, Training, and the Hypothalamic-Pituitary-Gonadal Axis in Men and Women. Front. Horm. Res..

