# Supplementary material for: Association of the Dietary Approaches to Stop Hypertension, Physical Activity, and Their Combination with Semen Quality: A Cross-Sectional Study
Source: Nutrients. 2019 Dec 22;12(1):39. doi: 10.3390/nu12010039 (PMC7019803; doi:10.3390/nu12010039)
Supplement: Supplementary File 1 [file nutrients-12-00039-s001.pdf]

Association of the Dietary Approaches to Stop Hypertension, Physical Activity and Their Combination with Semen Quality: A Cross-Sectional Study

Anna Danielewicz [1,*], Jakub Morze [1,2], Mariusz Przybyłowicz [3,4] and Katarzyna Eufemia Przybyłowicz [1]

1 Department of Human Nutrition, Faculty of Food Sciences, University of Warmia and Mazury in Olsztyn, ul. Słoneczna 45F, 10-718 Olsztyn, Poland; anna.danielewicz@uwm.edu.pl (A.D.); jakub.morze@uwm.edu.pl (J.M.); katarzyna.przybylowicz@uwm.edu.pl (K.E.P.);

2 Department of Cardiology and Cardiac Surgery, School of Medicine, University of Warmia and Mazury in Olsztyn, al. Warszawska 30, 10-082 Olsztyn, Poland;

3 Department of Oncological Gynecology, Provincial Specialist Hospital in Olsztyn, Żołnierska 18, 10-561 Olsztyn, Poland; mprzybylowicz@wp.pl

4 Center of Gynecology, Endocrinology and Reproductive Medicine Artemida in Olsztyn; Jagiellońska 78, 10-229 Olsztyn, Poland;

* Correspondence: anna.danielewicz@uwm.edu.pl; Tel.: +48-89-524-55-12

SUPPLEMENTARY MATERIALS

Table S1. List of components of the DASH score and their description.

**Score component**	Maximum score	Standard for maximum score	Standard for minimum score of 0	Food groups' description
Grains				
Total	5	≥6 servings per day	0 servings per day	light wheat, rye, wheat-rye bread, toasted bread, bread rolls, butter rolls and croissants, French croissants, pastries, pasta, rice, highly cleaned cereals, uncooked milk additives (e.g. muesli, cornflakes)
High fiber	5	≥50% of daily servings	0% of daily servings	wholemeal rye bread, with grains, pumpernickel, graham, buckwheat
Vegetables	10	≥4 servings per day	0 servings per day	broccoli, Brussels sprouts, fresh and sour cabbage, cauliflower, carrots, peppers, various salads, leeks, tomatoes, tomato and vegetable juice, red beets, onions, fresh and pickled cucumbers, root celery, sweetcorn, radish, mixed salad and mixed vegetable salad, potatoes and potato dishes
Fruit	10	≥4 servings per day	0 servings per day	apples, pears, plums, strawberries, cherries, bananas, oranges, grapefruits, kiwi, peaches, grapes, currants, berry fruits, juices: apple, orange, grapefruit, currant, other juices, multi-fruit
Dairy				
Total	5	≥2 servings per day	0 servings per day	milk, milk soups, milk drinks, yogurt, kefir, buttermilk, various creams, various cottage cheese, flavored and natural cottage cheese, rennet and processed cheese, spreads
Low-fat	5	≥75% of daily servings	0% of daily servings	low-fat milk, low-fat milk drinks, yogurt, kefir, buttermilk, various cottage cheese, flavored and natural cottage cheese, spreads
Meat, poultry, fish, eggs	10	≤2 servings per day	≥4 servings per day	sausages, luncheon meat, frankfurters, high-quality poultry and pork-beef sausages, liver, black pudding, headcheese, pates, bacon, pork, beef, veal, chicken meat from chicken, chicken, duck, turkey, fish (smoked, marinated, with sour cream, in oil, canned, fried, cooked), seafood, eggs mixed meat dishes with additives (e.g. bigos, stuffed cabbage rolls, hot dogs)
Nuts, seeds, legumes	10	≥4 servings per week	0 servings per week	nuts and seeds (various), peanut butter, chocolate-nut cream, green beans, green peas, beans, peas, lentils, broad beans
Fats, oils	10	≤3 servings per day	≥6 servings per day	butter, soft and hard margarine, oil, mayonnaise, dressings, lard, fat added to dishes
Sweets	10	≤5 servings per week	≥10 servings per week	sugar, honey, biscuits, cream cakes, shortbread, shortbread, with fruit, yeast dough, cheesecakes, donuts, poppy seed cake, chocolate, ice cream, pudding

Servings were presented for 2000 kcal/d. DASH - Diet Approaches to Stop Hypertension. Food groups description was based on Polish FFQ [18, 19].

Scheme S1. Matrix of the DASH diet and physical activity tertile (T) distribution.

		DASH diet		
		T1 (low)	T2 (moderate)	T3 (high)
Physical Activity	T1 (low)	**low**	**low**	**moderate**
	T2 (moderate)	**low**	**moderate**	**moderate**
	T3 (high)	**moderate**	**moderate**	**high**

Table S2. Diet and physical activity characteristics among tertiles of DASH score and physical activity.

Variable	Total	DASH			*P*	Physical activity			*P*
		T1	T2	T3		T1	T2	T3	
n	207	62	75	70		68	68	71	
**Dietary intake (servings/day)[1]**									
Total grains	2.9 (2.2; 4.0)	2.9 (1.8; 4.0)	2.8 (2.1; 3.7)	3.1 (2.6; 4.6)	0.050	3.2 (2.3; 4.4)	3.0 (2.1; 4.1)	2.8 (2.1; 3.6)	0.157
High-fiber grains [2]	17.5 (4.9; 37.3)	7.3 (2.5; 20.9)	13.8 (6.3; 38.2)	32.5 (16.3; 56)	<0.001	17.1 (6.4; 37.6)	17.6 (4.7; 35.7)	18.2 (4.5; 40.4)	0.930
Vegetables	2.6 (1.5; 3.6)	1.6 (0.9; 2.8)[a]	2.2 (1.5; 2.8)[b]	3.8 (2.8; 5.2)[ab]	<0.001	2.4 (1.3; 3.5)	2.5 (1.5; 3.6)	2.7 (1.7; 3.8)	0.582
Fruit	1.1 (0.6; 1.9)	0.6 (0.4; 1.4)[a]	1.0 (0.6; 1.6)[b]	1.8 (1.1; 2.6)[ab]	<0.001	0.9 (0.5; 1.4)	1.2 (0.6; 2.1)	1.2 (0.6; 1.9)	0.087
Total dairy	3.0 (1.8; 5.1)	2.5 (1.6; 4.3)	2.9 (1.8; 4.9)	3.5 (2.1; 5.2)	0.267	3.4 (2.2; 5.2)	2.6 (1.4; 4.9)	2.9 (1.8; 5.0)	0.149
Low-fat dairy [2]	76.2 (58.9; 87.2)	69.3 (50.7; 83.8)[a]	74.3 (59.3; 85.1)	80.7 (66.6; 89.4)[a]	0.044	76.5 (56.4; 89.6)	72.7 (53.5; 83.9)	77.3 (62.5; 89.5)	0.193
Meat, fish, eggs	3.4 (2.5; 4.5)	3.8 (2.6; 5.1)	3.2 (2.4; 3.9)	3.5 (2.9; 4.7)	0.045	3.7 (2.8; 4.7)	3;0 (2.4; 4.2)	3.5 (2.5; 4.5)	0.056
Nuts, seeds legumes	1.5 (0.6; 3.1)	0.8 (0.4; 1.6)[a]	0.9 (0.4; 1.8)[b]	3.7 (1.7; 5.7)[ab]	<0.001	1.3 (0.6; 3.0)	1.5 (0.7; 3.4)	1.6 (0.6; 3.0)	0.823
Fats, oils	2.2 (1.3; 3.0)	2.7 (2.0; 3.7)[ab]	2.1 (1.3; 2.8)[a]	1.9 (1.2; 2.9)[b]	0.001	2.4 (1.5; 3.3)	2.2 (1.4; 3;0)	2.1 (1.3; 2.9)	0.508
Sweets	4.9 (2.3; 7.9)	7.2 (4.3; 10.9)[ab]	3.9 (2.1; 6.5)[a]	4.9 (2.2; 6.7)[b]	<0.001	5.3 (2.6; 8.0)	4.6 (1.8; 7.3)	4.9 (2.9; 8.2)	0.386
**Physical activity**									
Total (MET-min/wk)	6827 (4198; 10032)	7185 (4575; 10860)	6075 (3540; 9530)	6942.3 (4323; 10350)	0.598	3109 (2055; 4178)	6732 (5865; 7656)	12258 (9996; 15264)	<0.001
Total time (min/wk)	153 (75; 342)	153 (63; 291)	116 (61; 287)[a]	205 (100; 432)[a]	0.026	77 (44; 124)	153 (90; 265)	291 (160; 486)	<0.001
Vigorous (min/wk)	12 (0; 42)	15 (1; 36)	11 (0; 36)	17 (2; 53)	0.167	1.5 (0; 15)	6 (0; 19.5)[a]	48 (18; 117)[a]	<0.001
Moderate (min/wk)	42 (15; 124)	36 (12; 116)	36 (12; 99)[a]	72 (25; 188)[a]	0.037	16.5 (6; 40)[ab]	70 (24; 134)[a]	81 (33; 192)[b]	<0.001
Walking (min/wk)	48 (15; 129)	45.5 (15; 92)	36 (14; 93)	53.5 (23; 168)	0.185	32 (10; 65)[a]	53 (25; 92)	48 (18; 117)[a]	0.001
Sedentary time (h/d)	2.5 (1.4; 4.1)	2.9 (1.8; 4.5)[a]	2.8 (1.5; 4.3)	1.8 (1.1; 3.9)[a]	0.035	3.0 (1.3; 4.8)	2.7 (1.5; 3.8)	2.2 (1.4; 3.9)	0.454
Sleeping (h/d)	7.0 (6.0; 8.0)	7.0 (6.5; 8.0)	7.0 (6.0; 8.0)	7.0 (6.0; 7.5)	0.069	7.0 (6.0; 8.0)	7.0 (6.8; 8.0)	7.0 (6.0; 8.0)	0.363

Data are presented as median and interquartile range (IQR). Dash- diet Approaches to Stop Hypertension, MET – metabolic equivalent of task. [1] servings per day adjusted for 2000 kcal. [2] percentage of daily servings. *P* for continuous variables were derived from Kruskal–Wallis test ([a,b] Dunn post-hoc test presented differences between pairs of tertiles).
